# Using artificial intelligence to generate medical literature for urology patients: a comparison of three different large language models

**DOI:** 10.1007/s00345-024-05146-3

**Published:** 2024-07-29

**Authors:** David Pompili, Yasmina Richa, Patrick Collins, Helen Richards, Derek B Hennessey

**Affiliations:** 1 School of Medicine, University College Cork, Cork, Ireland; 2 Department of Urology, Mercy University Hospital, Cork, Ireland; 3 Department of Clinical Psychology, Mercy University Hospital, Cork, Ireland

**Keywords:** Artificial intelligence (AI), Large language model (LLM), Patient information leaflet, ChatGPT, Google bard, Patient education

## Abstract

**Purpose:**

Large language models (LLMs) are a form of artificial intelligence (AI) that uses deep learning techniques to understand, summarize and generate content. The potential benefits of LLMs in healthcare is predicted to be immense. The objective of this study was to examine the quality of patient information leaflets (PILs) produced by 3 LLMs on urological topics.

**Methods:**

Prompts were created to generate PILs from 3 LLMs: ChatGPT-4, PaLM 2 (Google Bard) and Llama 2 (Meta) across four urology topics (circumcision, nephrectomy, overactive bladder syndrome, and transurethral resection of the prostate). PILs were evaluated using a quality assessment checklist. PIL readability was assessed by the Average Reading Level Consensus Calculator.

**Results:**

PILs generated by PaLM 2 had the highest overall average quality score (3.58), followed by Llama 2 (3.34) and ChatGPT-4 (3.08). PaLM 2 generated PILs were of the highest quality in all topics except TURP and was the only LLM to include images. Medical inaccuracies were present in all generated content including instances of significant error. Readability analysis identified PaLM 2 generated PILs as the simplest (age 14–15 average reading level). Llama 2 PILs were the most difficult (age 16–17 average).

**Conclusion:**

While LLMs can generate PILs that may help reduce healthcare professional workload, generated content requires clinician input for accuracy and inclusion of health literacy aids, such as images. LLM-generated PILs were above the average reading level for adults, necessitating improvement in LLM algorithms and/or prompt design. How satisfied patients are to LLM-generated PILs remains to be evaluated.

## Introduction

The advent and improvement of large language models (LLMs) and artificial intelligence (AI) generated information and content presents many exciting avenues by which clinical and non-clinical workloads may be reduced or improved [1]. LLMs are classified as generative AIs, which are trained to understand patterns and relationships between words and apply them to produce human-like responses to a given prompt [2]. Popular LLMs developed since 2022 include: OpenAI’s Generative Pre-Trained Transformer 4 (ChatGPT-4), Google’s Pathways Language Models 2 (PaLM 2) and Meta’s Large Language Model Meta AI (Llama 2).

While there exist numerous potential applications for AI in healthcare, the adaptive capabilities of generative AIs enable them to tailor the language, content, and style of their outputs, thereby potentially aiding in the communication of complex medical information to patients. Emerging evidence suggests that LLMs possess the capacity to produce empathetic responses to patients’ questions, which may be deemed preferable compared to physician-made responses [3–8]. Additionally, the medical accuracy of ChatGPT-4 and other LLMs is a topic of active research across many disciplines. In the context of urology, studies have shown both strong [9, 10] and poor [11] performance in providing evidence-based answers to short questions and clinical vignettes. However, the potential utility of LLMs in generating extended-form information for patients with urological conditions and procedures remains to be explored. Furthermore, existing literature primarily focuses on ChatGPT, leaving a gap to investigate how other novel LLMs perform in comparison.

This study aims to explore the ability of multiple mainstream LLMs (ChatGPT-4, PaLM 2, and Llama 2) to generate accurate patient information leaflets (PILs) on urological topics. In addition, given the growing importance of information accessibility for patient populations with varying degrees of education and literacy, we also investigated the readability of PILs generated by each LLM.

## Methods

### Patient information leaflet generation

This study was conducted in November 2023, ethical approval was granted by the Social Research Ethics Committee (SREC) of University College Cork Medical School. Four common surgeries and conditions were selected for this study, including circumcision, nephrectomy, overactive bladder syndrome (OAB) and transurethral resection of the prostate (TURP). These were chosen to capture a fair representation of the medical and surgical aspects of urology, encompassing the spectrum from benign to malignant surgery, simple to complex procedures and disease specific information. Three of the most popular LLMs were then selected for assessment, based on popularity and access, including OpenAI’s ChatGPT-4, Meta’s Llama 2, and Google’s PaLM 2 (Google Bard). A comprehensive prompt was written for each condition asking each LLM to generate a medically accurate PIL that was understandable to a layperson. The prompts were structured as follows: “Imagine you are a panel of experienced urologists and clinicians tasked to develop a patient information leaflet for (selected procedure/condition). Please ensure that the leaflet is medically accurate and based on current best practices/guidelines for urology. Please use clinical terminology while ensuring the leaflet is understandable to a layperson. Be reassuring and include images/diagrams where applicable”. Additional instructions within the prompt included using the headings provided in the associated leaflet produced by the European Association of Urology and including as much information as possible under each sub-heading. Examples of such instructions were “include all benefits, risks and potential complications of the procedure” and “include descriptions of what to expect both pre- and post-operatively and the steps patients can take to be active players in their care towards optimising patient outcomes.“. This prompt was tested multiple times in fresh sessions to assess for variability in LLM output. As there was minimal variability, a fresh session was then used and the first PIL provided by topic was recorded for subsequent scoring and readability analysis.

### PIL quality scoring

PILs were evaluated across 20 criteria using a previously developed quality checklist [12] using a 5-point Likert scale (0 = not applicable, 1 = strongly disagree, 2 = disagree, 3 = neither agree nor disagree, 4 = agree, 5 = strongly agree). The mean total quality checklist score was calculated by summing the 20 checklist items and dividing by 20.

PILs were copied with their original formatting as per the output by each LLM into separate Microsoft Word documents and labelled as version A, B or C. PIL scoring was undertaken in a single-session quality consensus meeting by a panel that was blinded to which LLM generated each PIL. The panel consisted of clinicians with varying degrees of urology training: three interns, three junior residents, three senior residents, and one consultant urologist. Each PIL in turn was read, discussed, and rated by consensus of the panel. In each evaluation, to reach consensus, panel members provided a score for each checklist criterion (Table 1) followed by active discussion of the pertinent points. Where disagreements occurred, the average score was recorded.

**Table 1:** Consensus scores for each LLM-generated PIL based on checklist quality criteria adapted from Sustersic et al. 2017 [12]

Checklist Criteria	Circumcision			Nephrectomy			Overactive Bladder Syndrome			Transurethral Resection of the Prostate		
	ChatGPT-4	Llama 2	PaLM 2	ChatGPT-4	Llama 2	PaLM 2	ChatGPT-4	Llama 2	PaLM 2	ChatGPT-4	Llama 2	PaLM 2
1. Based on the latest evidence-based medicine	4	4	5	2	1	1	4	4	5	2	4	2
2. Declares the objectives of the PILs (writer’s intention)	4	4	4	2	4	2	4	5	4	3	4	3
3. Explains causes, consequences, the usual course of the condition/disease	4	3	5	2	3	4	4	4	4	3	4	3
4. Explains the benefit/risks of a treatment, if any	5	4	5	3	3	4	3	3	3	2	5	3
5. Advice on who, when and where to consult	5	5	5	1	1	1	5	5	5	4	5	5
6. Advice on “what to do”: lifestyle recommendations, surveillance	4	5	4	3	4	4	5	5	5	3	4	5
7. Takes into account the patient’s needs according to the literature	3	3	3	3	3	3	4	4	4	3	3	3
8. Written so that it personally addresses the reader, targeted, culturally appropriate	5	5	5	3	3	3	3	3	3	3	3	3
9. Contains easy-to-understand illustrations, diagrams or photographs	1	1	5	1	1	5	1	1	5	1	1	5
10. Names the person who wrote the leaflet and their position. States date of writing and/or last update	1	1	1	1	1	1	1	1	1	1	4	1
11. Gives references to sources of the information	1	1	5	1	1	2	1	5	3	1	1	4
12. Avoids advertising or pharmaceutical brand names, uses generic names	5	5	5	5	5	5	5	5	5	5	5	5
13. Favours patient interaction through questions	3	3	3	3	4	4	3	3	4	4	4	4
14. Short format	5	5	4	4	4	4	5	2	5	4	4	4
15. Layout of information structured, presented in a logical order (paragraphs and titles)	5	5	5	4	5	5	2	4	4	5	5	5
16. Not too compact, simple presentation, avoiding colour overload in drawings and boxes	3	3	3	3	3	3	3	3	3	3	3	3
17. Simple vocabulary (words or groups of words)	4	4	4	3	4	4	2	2	3	2	3	2
18. Simple syntax (i.e. short sentences and active tense, active sentences)	3	3	2	4	4	3	4	2	3	2	2	2
19. Standard font (Arial, Times) avoiding small size (10 minimum)	4	4	4	4	4	4	4	4	4	4	4	4
20. Use of % to express frequencies, especially for risk perception	2	2	2	2	2	2	2	2	2	2	2	2
**Mean (SD)**	**3.55** (1.36)	**3.5** (1.36)	**3.95** (1.2)	**2.7** (1.14)	**3** (1.34)	**3.2** (1.29)	**3.25** (1.34)	**3.35** (1.31)	**3.75** (1.09)	**2.85** (1.19)	**3.5** (1.2)	**3.4** (1.2)

Llama 2 = Meta, PaLM 2 = Google Bard

### Readability assessment

Following the quality consensus meeting, each LLM-generated PIL was assessed for reading difficulty using the Average Reading Level Consensus Calculator so as not to influence the scoring by the panel. This online calculator (freely available at https://readabilityformulas.com/calculator-arlc-formula.php) takes the average of 7 popular readability formulas (Automated Readability Index, Flesch Reading Ease, Gunning Fog Index, Flesch-Kincaid Grade Level, Coleman-Liau Readability Index, SMOG Index, Linsear Write Readability Index) and produces a difficulty score based on grade levels ranging from extremely easy (first grade, age 6–7) to extremely difficult (college graduate, age 23+).

### Statistical analysis

Statistical analysis was performed using GraphPad Prism for Windows, version 10. Descriptive statistics were used to describe the data. A one-way ANOVA was performed by urological topic to assess for differences between PILs produced by each LLM.

## Results

### PIL quality scores across LLMs

**Fig. 1:**
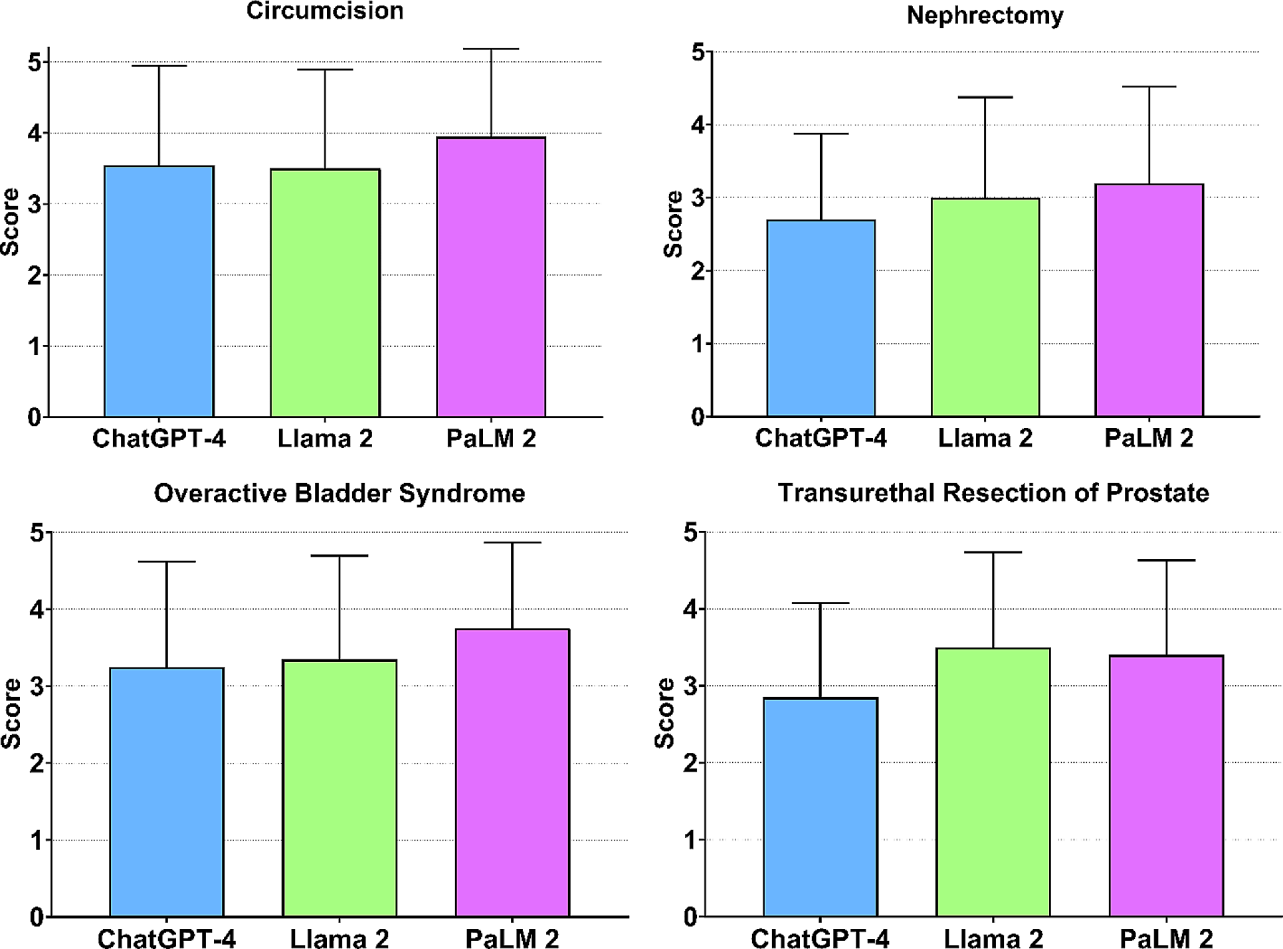
Mean total quality scores for each LLM generated PIL. Error bars represent standard deviation. There were no statistically significant differences within topic by one-way ANOVA. Llama 2 = Meta, PaLM 2 = Google Bard

PILs generated by PaLM 2 had the highest overall average quality score (3.58), followed by Llama 2 (3.34) and ChatGPT-4 (3.08). Overall, Google’s PaLM 2 generated PILs scored higher than Llama 2 and ChatGPT-4 PILs in all topics except TURP. In this case, Llama 2 achieved the highest mean score (Fig. 1). Circumcision PILs achieved the highest mean quality score of the 4 leaflets generated by PaLM 2 (3.95) and ChatGPT-4 (3.55). In the case of Llama 2, TURP and circumcision PILs achieved the highest quality mean score (3.5) (Fig. 1). There were no statistically significant differences in quality scores observed between the PILs generated by each LLMs across topics (Table 1).

### Medical accuracy of LLM-generated PILs

The medical accuracy of the PILs was assessed using scores from the evidence-based medicine criterion (item 1 of the quality checklist, Table 1). The OAB and circumcision PILs had no major errors and contained appropriate evidence-based information. Nephrectomy PILs scored the lowest across LLMs, with considerable errors and inaccurate information (Table 1). This included citing “kidney stones” (PaLM 2) or “an enlarged prostate” (Llama 2) as indications for nephrectomy.

In terms of the LLM performance, the mean evidence-based medicine checklist criterion scores for PaLM 2 and Llama 2 were the same across PILs at 3.25, outperforming ChatGPT-4’s mean score of 3 (Table 1).

### Inclusion of images

PaLM 2 was the only LLM to include images in its outputs. These images were taken from online web pages and included topic-specific anatomical diagrams or graphics illustrating aspects of the surgical procedure. While they did not include images, ChatGPT-4 and Llama 2 indicated where they would have included a figure (e.g. “Insert diagram showing TURP procedure, prostate gland, urethra and bladder.”), and briefly described in text what that figure would be showing to the reader (e.g. “Diagram: Illustration of the male urinary system showing the location of the prostate gland and the narrowing of the urethra due to BPH”).

### Readability assessment and word count

PILs generated by PaLM 2 were found to be the most readable, corresponding to a 9th Grade level (ages 14–15). Llama 2 PILs were the most difficult to read, representing an 11th grade (ages 16–17) level. Llama 2 PILs were consistently the longest, with two PILs exceeding 1000 words each. The OAB Llama 2 PIL was the longest at 2037 words, representing an almost 3-fold length increase compared to the next longest OAB PIL (Table 2).

**Table 2:** Average reading level consensus calculator scores and word count of PILs

	ChatGPT-4	Llama 2	PaLM 2
**Circumcision PIL**			
Reading Difficulty	Fairly Difficult	Somewhat Difficult	Somewhat Difficult
Grade Level	11	10	10
Age Range	16–17	15–16	15–16
Word Count	571	1096	887
**Nephrectomy PIL**			
Reading Difficulty	Somewhat Difficult	Difficult	Slightly Difficult
Grade Level	10	12	9
Age Range	15–16	17–18	14–15
Word Count	635	959	733
**Overactive Bladder Syndrome PIL**			
Reading Difficulty	Somewhat Difficult	Difficult	Slightly Difficult
Grade Level	10	12	9
Age Range	15–16	17–18	14–15
Word Count	709	2037	612
**Transurethral Resection of the Prostate PIL**			
Reading Difficulty	Fairly Difficult	Fairly Difficult	Slightly Difficult
Grade Level	11	11	8
Age Range	16–17	16–17	13–14
Word Count	637	914	767

Llama 2 = Meta, PaLM 2 = Google Bard

## Discussion

Integrating LLMs into healthcare settings holds the promise of improving how information is communicated to patients. LLMs such as ChatGPT-4, PaLM 2 (Google), and Llama 2 (Meta) have demonstrated capabilities in understanding, summarizing, and generating content. This study is the first to compare different LLMs in generating PILs in urology. Examining the LLM-generated PILs provides insights into the potential benefits, challenges, and necessary considerations for implementing LLMs in healthcare communication.

Our results reveal variations in the performance of different LLMs in generating PILs for urological topics. Among the three LLMs assessed, PaLM 2 emerges as the superior LLM, achieving the highest overall scores on the PIL checklist criteria. This suggests that PaLM 2’s outputs were generally perceived as more comprehensive and aligned with the quality checklist compared to ChatGPT-4 and Llama 2. PaLM 2 was the only LLM to incorporate images into its PILs, potentially enhancing understanding. However, the study also highlights an important caveat: the varying degrees of error in the medical accuracy of LLM-generated content. Thus, it is crucial to acknowledge the need for clinician oversight to ensure the accuracy of information provided by LLMs, especially in the context of providing patient information.

A number of studies have focused on the capabilities of ChatGPT-4 in the context of patient education [10, 13], in addition to its limitations [14]. In our study, ChatGPT-4 generated PILs had the lowest quality ratings amongst the three LLMs assessed. This underscores the importance of empirical testing to evaluate LLMs in a given context as their performance may not always align with expectations based on their utility in other applications.

In addition to evaluating the quality of the PILs, we conducted a readability analysis of their content. All PILs generated by the 3 LLMs examined in our study exceeded average literacy levels of Americans, which is suggested to be at the 7th to 8th grade level (12 to 14 years old) [15]. This finding is commensurate with previous studies demonstrating that the quality and readability of ChatGPT-4 responses had heightened complexity, surpassing optimal health communication readability [16], and that ChatGPT-4 generated materials were less accessible, with longer and more complex responses compared to traditional patient education materials [17]. Moreover, a recent report comparing 5 LLMs echoed our results, finding Google Bard (PaLM 2) to produce the most readable information [18], albeit still in excess of 7th to 8th grade level as in our study. Altogether, these data underscore that while LLMs can automate the generation of content, it is essential to balance providing comprehensive information and ensuring readability and thus accessibility for effective patient communication.

Interestingly, our study suggests that the complexity of medical topics may significantly influence the quality of generated PILs. The contrast in scores between PILs on circumcision and nephrectomy suggests a potential advantage in focusing on simpler subjects for effective PIL generation. Moreover, examining nephrectomy as a broad topic highlights that a more specific focus, such as a particular type of nephrectomy, may have yielded more targeted, accurate and informative content.

It is also prudent to consider the ease of use for each LLM interface. Both ChatGPT-4 and PaLM 2 had similarly accessible and simple online interfaces. However, Meta does not provide an online interface for Llama 2; instead, it requires downloading or use of a third-party online interface not produced by Meta, thus providing a barrier to entry for anyone seeking to use it. Understanding the user-friendliness and accessibility of these LLMs is crucial for practical implementation in healthcare.

While this study provides valuable insights, certain limitations should be acknowledged. Our evaluation was confined to a specific set of urological topics, and the findings relating to quality of LLM-generated PILs may not be generalizable to other medical specialties. Additionally, ongoing advancements in LLMs may influence outcomes, such as accuracy of information provided and inclusion of images, necessitating periodic reassessment. Although not explicitly tested in our study, future investigations might benefit from refining prompt design by opting for smaller, iterative, and sequential prompts to potentially enhance PIL quality and readability. Moreover, diversifying the PIL evaluation checklist to include specific medical accuracy and readability criteria could provide a more comprehensive assessment. Finally, we acknowledge the absence of patient involvement in the rating of the LLM-generated PILs. Further studies would be well placed including patient ratings of the acceptability and satisfaction with LLM-generated PILs. Despite these limitations, our study sheds light on the potential role of LLMs in generating PILs in the urology setting and thus alleviating aspects of the associated workload on healthcare professionals.

## Conclusion

In conclusion, this study provides valuable insights into the potential of LLMs, specifically ChatGPT-4, PaLM 2, and Llama 2, in generating PILs in urology. While these LLMs demonstrate the capacity to automate the creation of PILs, thereby reducing the workload of healthcare professionals, caution is warranted. Clinician input remains indispensable for ensuring medical accuracy and aligning readability levels with the intended lay patient audience. As the integration of LLMs in healthcare progresses, collaborative approaches that leverage both AI and human expertise will likely define the future landscape of patient medical communication.

## Data Availability

Available on request.
